# Oral microbiota in autoimmune polyendocrine syndrome type 1

**DOI:** 10.1080/20002297.2018.1442986

**Published:** 2018-02-26

**Authors:** Øyvind Bruserud, Huma Siddiqui, Mihaela Cuida Marthinussen, Tsute Chen, Roland Jonsson, Bergithe Eikeland Oftedal, Ingar Olsen, Eystein Sverre Husebye, Anette Bøe Wolff

**Affiliations:** ^a^ Department of Clinical Science, University of Bergen, Bergen, Norway; ^b^ K.G. Jebsen Center for Autoimmune Disorders, University of Bergen, Bergen, Norway; ^c^ Department of Oral Biology, Faculty of Dentistry, University of Oslo, Oslo, Norway; ^d^ Oral Health Centre of Expertise in Western Norway, Bergen, Norway; ^e^ Department of Clinical Dentistry, Faculty of Medicine, University of Bergen, Bergen, Norway; ^f^ Department of Microbiology, Forsyth Institute, Cambridge, MA, USA; ^g^ Department of Rheumatology, Haukeland University Hospital, Bergen, Norway; ^h^ Broegelmann Research Laboratory, Department of Clinical Science, University of Bergen, Bergen, Norway; ^i^ Department of Medicine, Haukeland University Hospital, Bergen, Norway

**Keywords:** APS-1, whole saliva, microbiota, bacteria, high throughput sequencing, pyrosequencing

## Abstract

**Background**: Autoimmune polyendocrine syndrome type-1 (APS-1) is a rare, childhood onset disease caused by mutations in the *Autoimmune Regulator* gene. The phenotypic expression is highly variable and includes disease manifestations in the oral cavity, including mucocutaneous candidiasis. Increasing evidence suggests a potential role of the skin, oral and gut microbiotas in the pathogenesis of autoimmunity. To date, no information exists regarding the oral microbiota in APS-1.

**Objective**: To assess the bacterial microbiota of whole saliva in APS-1 patients by using high throughput sequencing.

**Design**: Whole unstimulated saliva was collected from 10 APS-1 patients and 17 healthy controls and examined by high throughput sequencing of the hypervariable region V1-V2 of 16S rRNA using the 454 GS Junior system. Metastats (http://cbcb.umd.edu/software/metastats) was used to analyse the pyrosequencing reads.

**Results**: A reduction in the total number of bacterial genera and species was detected in APS-1 compared to healthy controls. The proportion of the major phyla Firmicutes was higher (60% vs 41%, *p* = 0.002) and Bacteroidetes lower (15% vs 28%, *p* = 0.007) in APS-1 compared to healthy controls. On the genus level, *Streptococcus* and *Gemella* were prevalent in APS-1.

**Conclusion**: Our findings indicate a significantly altered oral microbiota in APS-1.

Autoimmune polyendocrine syndrome type-1 (APS-1) or autoimmune polyendocrinopathy-candidiasis-ectodermal dystrophy (OMIM 240300) is a rare, monogenic, childhood onset disorder which is clinically defined by the presence of two of the three major disease components: primary adrenal insufficiency, hypoparathyroidism, and chronic mucocutaneous candidiasis (CMC) [1]. However, the clinical picture is highly variable and includes many minor disease components [2]. The *Autoimmune Regulator* (*AIRE)* gene is the disease-causing gene [3,4]. *AIRE* acts as a transcriptional regulator and is almost exclusively expressed in the thymus [5] where it orchestrates the process of negative selection of self-reactive T cells and contributes to the development of regulatory T cells (Tregs) [6,7]. All patients present autoantibodies against autoantigens expressed in the affected tissue [8] and/or against immune mediators such as interferon-omega (ω) and interleukin (IL)-22 [9,10]. Interestingly, circulating autoantibodies and *AIRE*-mutations can be found before development of clinical APS-1 [11,12] making the role of environmental triggers particularly relevant in the pathogenesis and phenotypic expression.

Increasing evidence indicates that the environment shapes the human immune system and accounts for its heterogeneity among individuals [13]. The oral, gut, and skin microbiotas could play a key role in the pathogenesis of systemic and organ-specific autoimmune diseases [14]. Most APS-1 patients develop disease components affecting the oral cavity; enamel hypoplasia and CMC are both common manifestations [9,15]. Also a Sjögren’s-like syndrome without extractable nuclear antigen autoantibodies has recently been described [15,16]. These oral manifestations probably interfere with the homeostasis of the oral microbiota. Furthermore, autoimmunity against defensins and other antimicrobial substances as observed in APS-1 could change the microbiota [17]. Reduced salivary flow rate changes the oral microbiota [18] and in a study of patients with severe Sjögren’s syndrome *Streptococcus salivarius, Neisseria pharyngis, Veillonella* species, and *Micrococcus mucilaginosus* were reduced and the number of *Staphylococcus aureus* and *Candida* species were increased compared to healthy controls [19]. Another recent study of primary Sjögren’s syndrome patients with normal salivary flow rate found that the number of bacterial genera and species was lower in patients, and concluded that saliva dysbiosis is a key characteristic of primary Sjögren’s syndrome [20]. Moreover, changes in the oral microbiota are found to be associated with several other diseases including squamous cell carcinoma, atherosclerosis, bacteraemia, and rheumatoid arthritis [21].

In APS-1, no information exists regarding the oral microbiota and only a few studies of the gut microbiota have been reported [17,22,23]. In this study, we characterized the bacterial profile in whole unstimulated saliva of patients by high throughput sequencing, a technique which recovers both cultivated and not-yet-cultivated bacteria, thus giving an in-depth overview of bacteria present.

## Materials and methods

### Patients and clinical data

A total of 10 APS-1 patients from 6 different families were included. All patients fulfilled the diagnostic criteria of APS-1. They were previously described in the Norwegian cohort [9,24,25] and included in our National Registry of Autoimmune Diseases. Three patients were excluded after an initial quality control. A detailed characterization of the seven remaining patients (five females and two males) is given in Table 1. The mean age was 32.3 years (range 10–64). All participants had their disease onset before the age of 8 years. Three patients presented the three major disease components and the mean number of disease components was five (range 4–7). Enamel hypoplasia was found in all patients. CMC was previously diagnosed in five patients and three had oral candidiasis at the time of sampling (Table 1). Disease-causing *AIRE-*mutations and autoantibodies against interferon-ω were present in all. All participants gave informed and written consent and the study was approved by The Regional Committee for Medical and Health Research Ethics for Western Norway.

**Table 1.** Characteristics of the APS-1 patients. The age at diagnosis for each disease component is written in parentheses. The age of onset denotes the age at which the first APS-1 main component appeared.

Pat. no.	Family no.	Sex	Age	Age of onset	Classic triad	Other manifestations	*AIRE*-mutations	Autoantibodies	Unstimulated saliva flow rate, saliva pH	Fungal load*	Other oral manifesta-tions
1	I	M	26	4	HP(4), CMC(9), AI(11)	E(11)	c.967_979del13/ c.967_979del13	21OH, IL17, IL22, INF-ω, MAGEB2, PDILT, SCC, TGM4, TH, TPH1	Pathological (<0.1 ml/min) pH 5.0	Positive. Cheilitis angularis.	Caries.
2	I	F	36	4	HP(4), CMC	E(14), AT(20), V(25)	c.967_979del13/ c.967_979del13	21OH, 17OH, AADC, GAD65, IL22, INF-ω, MAGEB2, NALP5, PDILT, SCC, SOX10, TH, TPH1	Normal (>0.1 ml/min) pH nd.	Negative.	–
3	II	F	10	7	PAI(7), HP(10), CMC	E, M	c.967_979del13/ c.967_979del13	21OH, 17OH, AADC, GAD65, IL22, INF-ω, MAGEB2, NALP5, SCC, TH, TPH1	Normal (>0.1 ml/min) pH 7.0	Negative.	–
4	III	M	64	7	CMC(7), HP(9), PAI(16)	V(17), Al(21), B12(63), E	c.769C>T/ c.769C>T	21OH, AADC, IL17, IL22, INF-ω, MAGEB2, SCC, SOX10, TGM4	Normal (>0.1 ml/min) pH 5.5	Positive. Cheilitis angularis.	Gingivitis. Caries.
5	IV	F	24	3	HP(3)	AT(24), E, M	c.967_979del13/ c.967_979del13	INF-ω, NALP5	Normal (>0.1 ml/min) pH 7.0	Negative.	Gingivitis.
6	V	F	25	2	CMC(2), HP(15)	E(24), Al, E	c.1163_1164insA/ c.1249_1950dupC	21OH, AADC, IL17, IL22, INF-ω, MAGEB2, NALP5, SOX10	Normal (>0.1 ml/min) pH 6.0	Positive. Cheilitis angularis.	Mucosal lesions.
7	VI	F	41	5	HP(5)	G(19), B12(35), M(39), E	c.934G>A (dominant) mutation)	AADC, GAD65, INF-ω, NALP5, PCA	Pathological (<0.1 ml/min) pH nd.	Negative.	Caries.

21OH, 21-hydroxylase; 17OH, 17-α-hydroxylase; AADC, aromatic l-amino acid decarboxylase; Al, alopecia; AT, autoimmune thyroiditis; B12, vitamin-B12 deficiency; CMC, candidiasis; E, enamel hypoplasia; G, hypogonadism; GAD65, glutamic acid decarboxylase 65-kDA isoform; HP, hypoparathyroidism; IL17, interleukin-17; IL22, interleukin-22; INF-ω, interferon-omega; M, malabsorption; MAGEB2, melanoma antigen B2; NALP5, NACHT leucine-rich-repeat protein 5; PAI, primary adrenocortical insufficiency; PCA, parietal cell autoantibodies; PDILT, protein disulphide isomerase-like testis expressed; SCC, side-chain-cleavage enzyme; SOX10, sex determining region Y-box 10; TGM4, transglutaminase 4; TPH1, tryptophan hydroxylase 1; TH, tyrosine hydroxylase; V, vitiligo; YoB, year of birth, nd; not done.

*Standard methods were used for culturing *Candida albicans*.

### Saliva sampling and sample processing

Whole unstimulated saliva was collected from patients (*n* = 10) and healthy controls (*n* = 17) with the same age and sex distributions as patients. Saliva samples were collected between 9 a.m. and 11 a.m., and participants were asked not to brush their teeth, eat, or drink for at least 2 h before sampling. No participants were regular smokers or had received antibiotics within the last month before sampling. Saliva pH was measured using a strip test (mColorpHast™ pH Test Strips, MilliporeSigma) and standard methods were used for  culturing *Candida albicans*. Unstimulated saliva flow rate (ml/min) was measured based on a collection period of 15 min (Table 1). Samples were immediately stored at −80°C until analysis.

Sample processing was done as previously described [20,26]. In summary, DNA was extracted from a 250 µl sample volume using the MasterPure™ DNA Purification kit (Epicentre, Illumina Company, Madison, WI) and was dissolved in 45 µl 1 × TE buffer. The 16S rRNA hypervariable region V1-V2 was amplified in three parallel PCRs and sequenced on a 454 GS Junior System (Roche, Branford, CT). Primer sequences and amplifications reactions are given previously [20]. DNA quality and concentrations were assessed with Bioanalyzer 2100 (Agilent, Santa Clara, CA) and Nanodrop 3300 Flurospectrometer (Thermo Scientific, Wilmington, DE), all within the range to perform high throughput sequencing.

### Bioinformatics analysis of sequence reads

Bioinformatics analysis of sequence reads was executed as described previously [20]. In brief, raw sequence reads were subjected to a species-level, reference-based taxonomy assignment especially designed for studying the human oral microbial community [20]. The set of 16S rRNA reference sequences previously published by Al-Hebshi et al. [27] and the NCBI 16S rRNA reference sequence set (ftp://ftp.ncbi.nlm.nih.gov/blast/db/16SMicrobial.tar.gz) were combined giving reference sequences representing a total of 1,151 oral and 12,013 non-oral species that were BLASTN-searched for each of the sequence reads. Unassigned reads were then screened for high-quality non-chimeras and subjected to *de novo* species-level operational taxonomy unit calling for potential novel species. The quantitative insights into microbial ecology pipeline software package version 1.9.1 [28] was used for down-stream analyses, including alpha and beta diversities. A statistical method introduced in Metastats (http://cbcb.umd.edu/software/metastats) was used to reveal significant differences between the microbiota of control saliva and APS-1 saliva. This method employs a false discovery rate to improve specificity in high complexity environments, and handles sparsely sampled features using Fisher’s exact test [29]. *p*-values ≤ 0.05 were considered significant and Bonferonni Correction for multiple testing was included.

## Results

### Sequence data

An overview of the sequence read counts in each analysis step is given in Supplemental Table 1.

### Composition of the salivary microbiota of APS-1 patients

**Figure 1.**
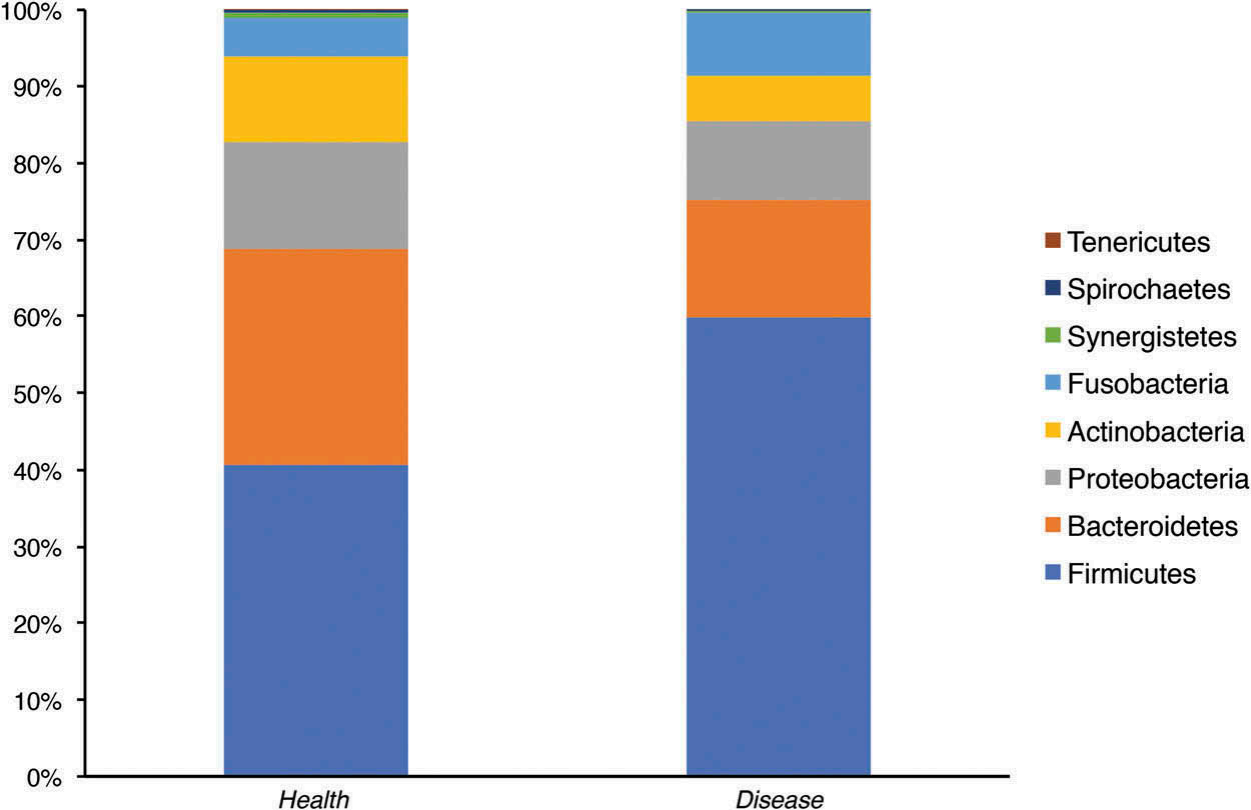
Bacterial phyla detected in APS-1 patients (*n* = 7) and control saliva (*n* = 17). Comparison of microbiota in APS-1 and healthy saliva determined by sequencing the hypervariable *16S rDNA* region V1-V2. Relative abundance of the different phyla in control and APS-1 samples is shown.

**Figure 2.**
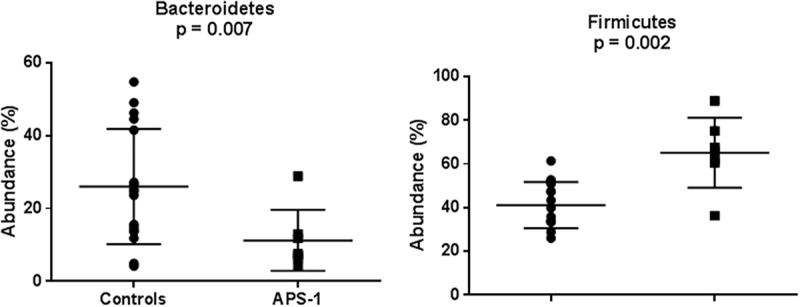
The relative abundances of the phyla Bacteroidetes and Firmicutes in saliva of healthy controls (*n* = 17) and APS-1 patients (*n* = 7) illustrated with boxplots. The lines indicate means and standard deviations.

Five different phyla were detected in APS-1 patients with DNA sequences predominately assigned to the phyla Firmicutes (60%), Bacteroidetes (15%), Proteobacteria (10%), Fusobacteria (8%), and Actinobacteria (6%). Table 2 gives an overview of the significantly different abundances of taxa from control saliva and APS-1 saliva by phyla, genera and species, respectively. In Figure 1, a comparison of the bacterial content of saliva in APS-1 and controls based on the sequencing of the hypervariable 16S rDNA region V1-V2 is presented. Saliva from APS-1 displayed a significantly increased relative abundance of Firmicutes (*p *= 0.002) and a lower frequency of Bacteroidetes (*p *= 0.007) compared with controls (Figure 2).

**Table 2.** Differences in abundances of taxa in APS-1 and control saliva. Significant (*p* ≤ 0.05) differences in relative abundance of taxa from control saliva and APS-1 saliva as estimated by Metastats http://cbcb.umd.edu/software/metastats. Differences significant after Bonferonni Correction for multiple testing are marked with *.

Taxon	APS-1 saliva (*n* = 7)	Control saliva (*n* = 17)	Metastat *p*-value
**Phyla**			
Firmicutes	0.65118 ± 0.02551	0.41118 ± 0.02572	0.002
Bacteroidetes	0.11271 ± 0.03162	0.26009 ± 0.03845	0.007
**Genera**			
*Streptococcus*	0.53448 ± 0.08523	0.18016 ± 0.01963	0.0001*
*Gemella*	0.01467 ± 0.00023	0.00253 ± 0.00062	0.036
*Prevotella*	0.05995 ± 0.01021	0.17357 ± 0.02970	0.001
*Veillonella*	0.04049 ± 0.00925	0.16254 ± 0.02760	0.0001*
*Neissera*	0.02065 ± 0.00965	0.05508 ± 0.01269	0.029
*Actinomyces*	0.00148 ± 0.00055	0.00673 ± 0.00159	0.002
*Megashaera*	0.00000	0.00631 ± 0.00287	0.027
*Lachnospiraceae*_ [G-3]	0.00059 ± 0.00033	0.00417 ± 0.00097	0.001
*Lachnoanaerobaculum*	0.00024 ± 0.00019	0.00123 ± 0.00033	0.008
*Eubacterium*_[XIVa][G-1]	0.00025 ± 0.00018	0.00109 ± 0.00031	0.019
*Ruminococcaceae*_[G-3]	0.00002 ± 0.00776	0.00119 ± 0.00448	0.016
*Bacteroides*	0.00000	0.00049 ± 0.00019	0.012
*Veillonella*	0.00000	0.00053 ± 0.00025	0.031
*Bacteroidetes*_[G-3]	0.00000	0.00032 ± 0.00010	0.002
*Peptostreptococcaceae_[*XI][G-4]	0.00000	0.00023 ± 0.00011	0.034
*Mitsuokella*	0.00000	0.00033 ± 0.00016	0.038
*Desulfovibrio*	0.00000	0.00405 ± 0.00040	0.018
*Catonella*	0.00000	0.00015 ± 0.00013	0.048
**Species**			
*Streptococcus* sp._str._C300	0.12958 ± 0.03003	0.03065 ± 0.00618	0.001
*Streptococcus* multispecies_spp24_2	0.11555 ± 0.05022	0.00877 ± 0.00349	0.031
*Fusobacterium nucleatum_*ss*_animalis*	0.02401 ± 0.01178	0.00083 ± 0.00038	0.046
*Gemella haemolysans*	0.01308 ± 0.00599	0.00132 ± 0.00047	0.047
*Capnocytophaga ochracea*	0.00112 ± 0.00035	0.00004 ± 0.00002	0.023
*Veillonella parvula_*group	0.03273 ± 0.00653	0.10745 ± 0.01904	0.0003*
*Prevotella melaninogenica*	0.02205 ± 0.00876	0.09752 ± 0.01762	0.0002*
*Veillonella atypica*	0.00776 ± 0.00389	0.05509 ± 0.01369	0.001
*Neisseria flavescens/subflava*	0.01723 ± 0.00873	0.04995 ± 0.01204	0.023
*Porphyromonas gingivalis*	0.00000	0.02849 ± 0.01465	0.048
*Prevotella pallens*	0.00059 ± 0.00036	0.02308 ± 0.00617	0.0004*
*Campylobacter concisus*	0.00133 ± 0.00083	0.01390 ± 0.00382	0.001
*Prevotella salivae*	0.00054 ± 0.00024	0.00848 ± 0.00293	0.006
*Prevotella veroralis*_nov_95.28%	0.00000	0.00723 ± 0.00287	0.010
*Solobacterium moorei*	0.00251 ± 0.00121	0.00638 ± 0.00154	0.045
*Megasphare micronuciformis*	0.00000	0.00631 ± 0.00287	0.023
*Atopobium parvulum*	0.00099 ± 0.00026	0.00579 ± 0.00223	0.028
*Lachnospiraceae*_[G-3] sp._oral_taxon_100_nov_83.29%	0.00049 ± 0.00032	0.00342 ± 0.00082	0.001
*Actinomyces* sp._Oral_Taxon_180	0.00043 ± 0.00022	0.00328 ± 0.00103	0.006
*Actinomyces odontolyticus*	0.00047 ± 0.00015	0.00247 ± 0.00062	0.001
*Prevotella* multispecies_sppn1_2_nov_82.34%	0.00027 ± 0.00019	0.00158 ± 0.00579	0.026
*Lachnoanaerobaculum orale*	0.00024 ± 0.00019	0.00123 ± 0.00332	0.008
*Eubacterium*_[XIVa][G-1] *saburreum*	0.00025 ± 0.00018	0.00109 ± 0.00313	0.016
*Tannerella forsythia*_nov_96.52%	0.00000	0.00095 ± 0.00311	0.002
*Aggregatibacter aphrophilus*	0.00000	0.00080 ± 0.00035	0.018
*Porphyromonas gingivalis*_nov_96.46%	0.00000	0.00079 ± 0.00037	0.026
*Leptotrichia* sp._oral_taxon_219_nov_83.33%	0.00021 ± 0.00019	0.00077 ± 0.00020	0.039
*Fretibacterium fastidiosum*	0.00000	0.00074 ± 0.00033	0.019
*Ruminococcaceae*_[G-3] sp._oral_taxon_366_nov_78.83%	0.00002 ± 0.00002	0.00054 ± 0.00015	0.001
*Veillonella* sp._oral_taxon_780_nov_82.14%	0.00000	0.00053 ± 0.00025	0.029
*Mogibacterium neglectum*	0.00010 ± 0.00008	0.00051 ± 0.00190	0.044
*Haemophilus parainfluenzae*_nov_96.89%	0.00000	0.00043 ± 0.00026	0.018
*Desulfovibrio* sp._oral_taxon_040	0.00000	0.00040 ± 0.00040	0.018
*Mitsuokella* sp._Oral_Taxon_H31_nov_78.38%	0.00000	0.00033 ± 0.00016	0.035
*Streptococcus constellatus*_nov_80.22%	0.00000	0.00032 ± 0.00013	0.008
*Porphyromonas gingivalis*_nov_91.05%	0.00004 ± 0.00004	0.00032 ± 0.00011	0.019
*Bacteroidetes*_[G-3] sp._oral_taxon_280_nov_92.66%	0.00000	0.00031 ± 0.00010	0.015
*Alloprevotella rava*_nov_86.47%	0.00000	0.00029 ± 0.00009	0.002
*Fretibacterium* sp._oral_taxon_359	0.00000	0.00025 ± 0.00016	0.029
*Prevotella* sp._oral_taxon_515_nov_80.56%	0.00000	0.00024 ± 0.00013	0.018
*Prevotella* sp._oral_taxon_515_nov_78.25%	0.00000	0.00023 ± 0.00016	0.029
*Peptostreptococcaceae*_[XI][G-4] sp._oral_taxon_369	0.00000	0.00022 ± 0.00011	0.031
*Catonella* sp._oral_taxon_164_nov_95.73%	0.00000	0.00015 ± 0.00014	0.048

**Figure 3.**
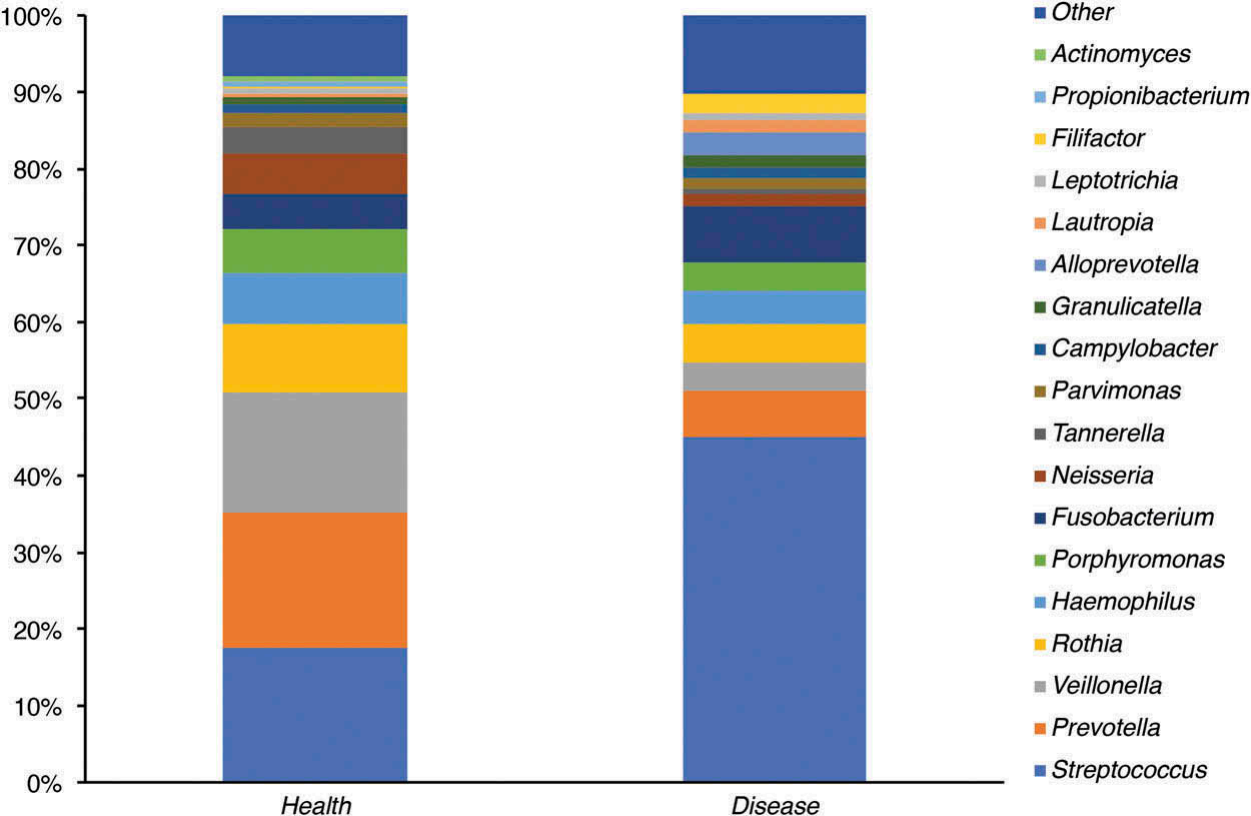
Bacterial genera detected in saliva from APS-1 patients (*n* = 7) and controls (*n* = 17). Groups designated as ‘Other’ represents minor groups classified. The *Y*-axis represents relative abundance. An increase in the genus *Streptococcus* in APS-1 saliva relative to control saliva is demonstrated.

**Figure 4.**
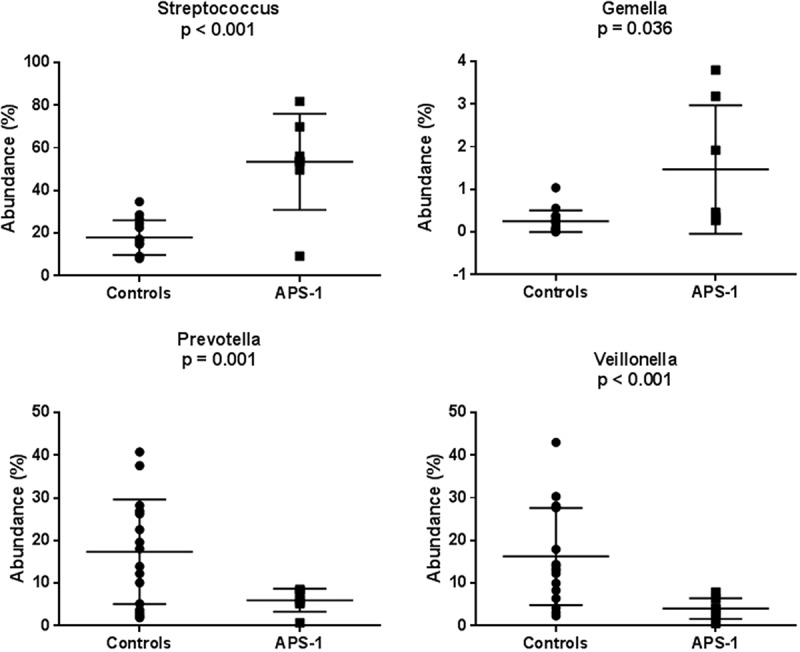
The relative abundances of the genera *Streptococcus, Gemella, Prevotella*, and *Veillonella* in saliva of healthy controls (*n* = 17) and APS-1 patients (*n* = 7) illustrated with boxplots. The lines indicate means and standard deviations.

A total of 64 bacterial genera were detected in APS-1 and 90 in healthy controls. Figure 3 gives the relative abundances of the 18 major genera found in APS-1 samples and controls. Moreover, a total of 18 genera showed significant differences in abundances (Table 2). Metastats analyses showed that the genera *Streptococcus* and *Gemella* were significantly higher in patients than controls (*p = *0.0001 and 0.036, respectively; Figure 4) whereas *Prevotella* and *Veillonella* were significantly higher in controls (*p = *0.001 and <0.001, respectively; Figure 4). Among the genera with significant difference in abundance, eight genera were absent from whole saliva of APS-1 patients (Table 2).

In APS-1 patients, the sequencing data revealed the most abundant species to be *Streptococcus* sp. str. C300, *Streptococcus* multispecies spp.24_2, *Streptococcus mitis, Streptococcus infantis*, and *Haemophilus parainfluenzae*. Among these, only *Streptococcus* sp. str. C300 and *Streptococcus* multispecies spp.24_2 were increased compared to controls (*p = *0.001, 0.032, respectively). The other species found more abundant in APS-1 patients were *Fusobacterium nucleatum* subsp. *animalis, Gemella haemolysans, Ruminococcaceae* [G-3] sp. Oral taxon 366, and *Capnocytophaga ochracea* (*p = *0.046, 0.047, <0.001, 0.002, respectively). The *Veillonella parvula* group and *Prevotella melaninogenica* were most abundant in controls. Table 2 shows all species with significant differences in abundance compared to control saliva and APS-1 saliva as estimated by Metastats.

### Species richness and diversity

**Figure 5.**
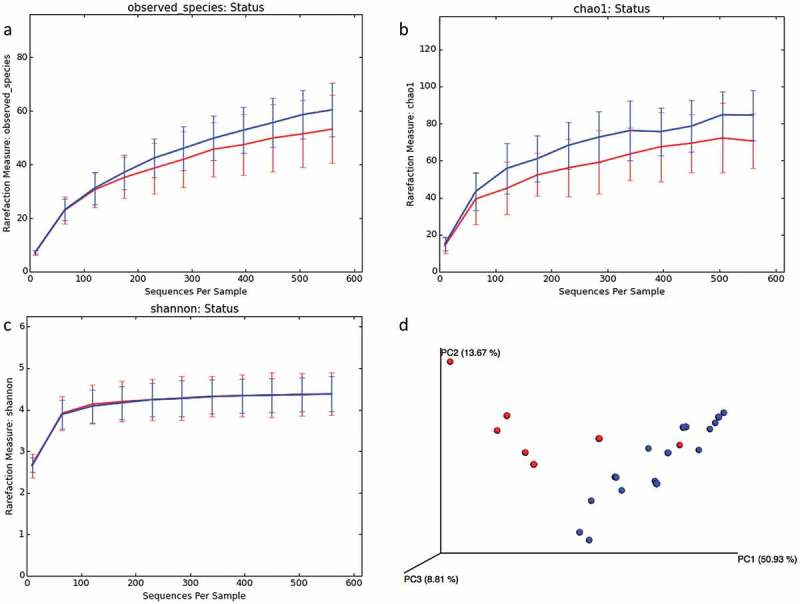
Comparison of microbial diversity in saliva samples of APS-1 patients (*n* = 7) and health samples (*n* = 17). Average rarefaction curves of APS-1 and health samples reported as (a) observed species, (b) Chao1 estimator, (c) Shannon index, (d) PCoA 3D plots of all samples with distances calculated using weighed normalized UniFrac matrix. Blue curves and dots represent health samples; and red represents APS-1.

Samples from healthy controls had a higher species richness and diversity than APS-1 samples. Figure 5(a) illustrates that the average species richness is higher in healthy subjects based on the observed species rarefaction curves Figure 5(b,c) gives the estimated species richness evaluated on the Chao1 matrix and the rarefaction curves based on the Shannon index, respectively showing an overall difference in alpha diversity, although the latter shows no difference between health and APS-1. However, the Shannon index measures both richness and abundance, hence the evenness of the community. Figure 5(c) therefore indicates that there is no significant difference between APS-1 and controls in terms of species evenness. Finally, Figure 5(d) gives a PCoA 3D plot of all samples with the distances calculated using weighed normalized UniFrac matrix, and clearly indicates that the two groups have distinct beta diversity. Taken together, these results show a richer diversity in controls compared to APS-1.

## Discussion

Increasing evidence suggests a potential role of the skin, oral, and gut microbiotas in the pathogenesis of autoimmunity [14]. Using high throughput sequencing and a comprehensive bioinformatics approach to analyse next generation 16S rDNA pyrosequencing reads, we here present hitherto undescribed significant differences in the composition of the salivary microbiota comparing APS-1 patients and healthy controls, indicating a possible contribution in pathogenesis and clinical expression.

The most striking differences were a higher portion of Firmicutes and a reduction of Bacteroidetes  in APS-1 patients. Similar findings were recently described in primary Sjögrens syndrome [20] and a reduced species diversity and an altered ratio between Firmicutes and Bacteroidetes were described in the intestinal microbiota in several autoimmune diseases [30–33]. However, Firmicutes represents a phylum where most bacteria have a Gram-positive cell wall including the oral genera *Streptococcus, Lactobacillus, Selenomonas, Clostridium*, and *Eubacterium*. The phylum Bacteroidetes is composed of Gram-negative bacteria. On a genus level, *Streptococcus* and *Gemella* were increased in APS-1. However, a reduction in the total number of bacterial genera was seen. Overall, these novel findings indicate a significant altered oral microbiota in APS-1.

This is the first report on the oral microbiota in APS-1, and only a few studies have investigated the gut microbiota in patients [17,22,23]. A comparative analysis of the intestinal microbiota of APS-1 patients with gastrointestinal manifestations showed significant enrichment of segmented filamentous bacteria [17], which are Gram-positive commensal bacteria with the potential to adhere to epithelial cells and induce T helper (Th) 17 responses. Another study reported that APS-1 patients develop early and sustained responses to gut microbial antigens reminiscent to Crohn’s disease [23] linked to defects in Tregs. Finally, products from commensal bacteria have the potential to indirectly regulate thymic *Aire* expression in mice [22]. Based on the above, this indicates that the microbiota contributes in shaping immunity in APS-1 although the molecular mechanisms are incompletely characterized.

Factors known to directly affect the immune system, and consequently, the risk of autoimmunity, such as genetics, gender, and diet may also exert their effects by modulating microbiota profiles and functions. Using animal models of experimental colitis [34] and arthritis [35] it was shown that Gram-negative bacteria, possibly through the TLR2/IL-10 axis, reduced inflammation [34], whereas Gram-positive bacteria contributed to a more severe disease [35]. In a mouse model of Sjögren’s disease, depletion of the intestinal microbiome worsened the ocular response to desiccation, while the overall severity of disease correlated with intestinal microbiome diversity [36]. Two recent characterizations of the oral microbiota in primary Sjögren’s syndrome demonstrated a significant shift in the oral microbiota of patients and reduced numbers of genera [20,37], suggesting a role of the oral microbiota in the pathogenesis. Interestingly, these findings are comparable to what we currently describe in APS-1.

CMC caused by *C. albicans* is the most common and earliest manifestation of APS-1 [2]. The clinical course varies from periodical to chronic and usually affects the oral mucosa [1,2,9]. To date, neutralizing autoantibodies against the Th17 cytokines IL-17A, IL-17F, and IL-22 are suggested to explain the impairment in mucosal immunity in APS-1 patients [10,38]. However, an important line of defence against oral CMC is the oral microbiota that prevents infections by their interplay with immune cells, nutrients, metabolic products and by secreting antagonistic molecules, which together balance local inflammatory responses [39]. *C. albicans* can form biofilms with many oral bacteria, including streptococci [40], which have synergistic or antagonistic influences on *C. albicans*. Noteworthy, recent work has highlighted the critical role of metabolic products from specific gut microbiota such as lactobacilli in priming IL-22 dependent mucosal immune responses by innate lymphoid cells via the aryl hydrocarbon receptor, which is fundamental for protection against uncontrolled local *Candida* expansion [41]. Further, a shift in salivary microbiota has been linked to the risk of oral cancer in selected groups of patients [42]. We found significantly increased abundance of streptococci in APS-1 saliva compared to healthy controls and several streptococci were among the most abundant species in APS-1 saliva. To speculate, an altered microbiota may change the profile of immune regulatory metabolic products, and thus, contribute in altering immunity in APS-1 patients. Still it remains unclear whether an altered microbiota causes disease manifestations or the altered microbiota is an effect of disease components.

The obvious drawbacks of this study are the low number of patients included and the heterogeneity within this group. Initially 10 patients were included but technical issues with the sequencing made us exclude 3 patients. We plan to include more patients trying to correlate the different taxa to clinical manifestations of APS-1 and calculate absolute microbial abundances using a quantitative polymerase chain reactions assay. The same approach could be used to characterize the skin and the gut microbiotas in APS-1.

In conclusion, the oral microbiota in APS-1 patients is altered compared to healthy controls and seems to be a characteristic of the syndrome. In general, the skin, oral, and intestinal microbiotas in APS-1 patients should be further investigated to reveal their potentially contribution in pathogenesis and phenotypic expression of the syndrome.

## Supplementary Material

Supplementary_Table_1.docx
