# Supplementary material for: Oral microbiota in autoimmune polyendocrine syndrome type 1
Source: J Oral Microbiol. 2018 Feb 26;10(1):1442986. doi: 10.1080/20002297.2018.1442986 (PMC5827717; doi:10.1080/20002297.2018.1442986)
Supplement: Supplementary_Table_1.docx [file ZJOM_A_1442986_SM2682.docx]

**Supplementary Table 1.**

Summary of the sequence read counts in each analysis step

| Total read counts | 126,968 |
| --- | --- |
| Total assigned reads | 81,153 |
| Assigned reads excluded due to < 10 reads per species | 1,478 |
| Total assigned reads used for analysis* | 79,675 |
| Reads assigned to single species | 69,400 |
| Reads assigned to multiple species | 3,608 |
| Reads assigned to novel species | 6,667 |
| Total unassigned reads (chimera, singletons and reads without BLASTN hits) | 45,815 |
| * Total assigned reads used for analysis is the sum of total reads assigned to unique, multiple, and novel species. The mean ± standard deviation of reads per sample used for analysis was 3320±1579. |  |
